# A simulation tool for better management of retinal services

**DOI:** 10.1186/s12913-018-3560-5

**Published:** 2018-10-04

**Authors:** Eren Demir, David Southern, Aimee Verner, Winfried Amoaku

**Affiliations:** 1 University of Hertfordshire, Hertfordshire Business School, Hertfordshire, UK; 2 Pathway Communications Ltd, Cambridge, UK; 3 Cambridge University Hospitals NHS Foundation Trust, Cambridge, UK; 4 Academic Ophthalmology, DCN, Faculty of Medicine, The University of Nottingham, Nottingham, UK

**Keywords:** Virtual clinics, Treat and extend, Retinal services, Discrete event simulation, Patient flow modelling

## Abstract

**Background:**

Advances in the management of retinal diseases have been fast-paced as new treatments become available, resulting in increasing numbers of patients receiving treatment in hospital retinal services. These patients require frequent and long-term follow-up and repeated treatments, resulting in increased pressure on clinical workloads. Due to limited clinic capacity, many National Health Service (NHS) clinics are failing to maintain recommended follow-up intervals for patients receiving care. As such, clear and robust, long term retinal service models are required to assess and respond to the needs of local populations, both currently and in the future.

**Methods:**

A discrete event simulation (DES) tool was developed to facilitate the improvement of retinal services by identifying efficiencies and cost savings within the pathway of care. For a mid-size hospital in England serving a population of over 500,000, we used 36 months of patient level data in conjunction with statistical forecasting and simulation to predict the impact of making changes within the service.

**Results:**

A simulation of increased demand and a potential solution of the ‘Treat and Extend’ (T&E) regimen which is reported to result in better outcomes, in combination with virtual clinics which improve quality, effectiveness and productivity and thus increase capacity is presented. Without the virtual clinic, where T&E is implemented along with the current service, we notice a sharp increase in the number of follow-ups, number of Anti-VEGF injections, and utilisation of resources. In the case of combining T&E with virtual clinics, there is a negligible (almost 0%) impact on utilisation of resources.

**Conclusions:**

Expansion of services to accommodate increasing number of patients seen and treated in retinal services is feasible with service re-organisation. It is inevitable that some form of initial investment is required to implement service expansion through T&E and virtual clinics. However, modelling with DES indicates that such investment is outweighed by cost reductions in the long term as more patients receive optimal treatment and retain vision with better outcomes. The model also shows that the service will experience an average of 10% increase in surplus capacity.

**Electronic supplementary material:**

The online version of this article (10.1186/s12913-018-3560-5) contains supplementary material, which is available to authorized users.

## Background

Hospital Eye Services in the United Kingdom (UK) National Health Service (NHS) have consistently had a low profile, despite providing a high volume of work. They account for nearly one in ten hospital outpatient appointments, as the second highest demand specialty with 5.95 million clinic attendances in 2009/10 in England and 7–8% of all surgical operations performed by the NHS. Furthermore, demand has risen by 25% over 7 years [1].

Amongst all ophthalmology clinic attendances, retinal vascular diseases are a leading cause of sight loss. The three specific causes of such visual loss are age related macular degeneration (AMD), diabetic retinopathy (DR), and retinal vascular occlusions (RVO). These conditions are of particular interest as their prevalence is rising in an increasingly ageing population, as AMD and RVO are associated with increasing age, and diabetes mellitus, occurs more frequently. AMD as a serious macular disorder, which is forecasted to rise from 513,000 in 2010 to 679,000 in 2020, an increase of 32% in just 10 years [2]. These patients may require monthly outpatient visits and treatments until disease stability is achieved, followed by implementation of various regimens including pro re nata (PRN) treatment, fixed interval dosing being the most popular until recently [3–6]. Emerging evidence suggests that outcomes are better with Treat and Extend (T&E) regimen [7–11]. However, these treatments and regimen are resource intensive, and require multi-disciplinary teams including photographers, optometrists, specialist nurses, and pharmacists, in addition to the ophthalmologist.

Given the frequency of treatment and monitoring visits (usually monthly), combined with increasing demand for services where the number of treatable patients potentially suitable for treatment have risen markedly, many NHS ophthalmology clinics are currently running at 100% capacity and some are failing to maintain the recommended follow-up and re-treatment intervals. Most of these clinics which are running at maximum capacity may not have scope for further expansion. They cannot cope with the current patient numbers, let alone allow for future increases in patient numbers.

It is clear that robust and long term retinal service delivery modelling is required across NHS clinics in order to determine the current and future needs of local populations, so that there is no future compromise in the standard of service provision, including follow-up (FU) intervals and quality of intravitreal treatment administration. Amoaku et al. (2012) [12] produced a comprehensive list of changes that can be made in order to meet existing demand and needs of patients with limited resources and capacity. In that publication, the authors shared a number of service re-design case studies, e.g., virtual clinics. Virtual clinics usually consist of non-mydriatic photos of the optic disc, visual fields, and visual acuity measurements carried out by a technician, stored, and later reviewed by an Ophthalmologist [13]. A virtual clinic can be within a hospital setting or in a location within the community. If it is located in the community then patients are shifted from a hospital setting where treatment (or a follow-up routine appointment) is provided out in the community, thus releasing the pressure on hospitals. A number of other authors have presented similar models (including virtual clinics) of dealing with capacity constraints [14–18].

Despite these efforts, the quantifiable impact of re-designing retinal services has never been studied. Whether planned service re-design will actually result in increased capacity, and if so how much cost improvement will result remains unknown. Furthermore, it is unknown as to how a change in one area of the service will impact on resources in another including activity (new and FU appointments), resource utilisation and costing and revenue.

The aims of this study are therefore: 1) to develop a discrete event simulation (DES) model, which captures the retinal service treatment pathways and service re-designs including virtual clinics; 2) Second, to determine possible and realistic policies, which could be implemented with regard to an increased use of virtual clinics combined with a new treatment regimen (known as treat and extend), and how these would affect hospital based treatment; 3) to evaluate the impact of the implementation of this service re-design on a number of operational and cost performance indicators relevant to the delivery of retinal services. This should provide an indication of the feasibility and scale of improvement, both operationally and patient outcomes, and allow health managers to make informed decisions with regard to the best ways to reconfigure the delivery of retinal services in the UK. This study was based on the retinal service of a mid-sized NHS hospital Trust in England.

## Methods

A unique version of a discrete event simulation model was developed for Cambridge University Hospitals NHS Foundation Trust in three stages. Firstly, the Trust’s patient level ophthalmology data was anonymised and analysed in greater detail. Secondly, the structure of the Trust’s intravitreal injection service as delivered at the time (mainly a prn regime) was established using management and clinician expertise. Finally, the combination of both sets of analysis and statistical forecasts were entered into the model. Using sophisticated statistical algorithms, we forecast activity by patient type (AMD, DR and RVO) broken down by new patients and follow-up (FU) visits. The simulation was then ran many times giving accurate information on the next twelve months of activity, resource utilisation and revenue/costing to provide baseline values. The baseline was then compared to any number of other alternative methods of delivering the intravitreal injection service. This enabled the Trust to explore the impact of service redesign options rapidly and with ease in order to identify the most effective solutions.

### The problem/hypothesis

In order to address the issue of increasing patient numbers and to demonstrate how the model could be used to identify service efficiencies, we explored the impact on the service by adopting the T&E regimen in combination with virtual clinics compared to the current regime of prn delivery as described in the SUSTAIN Study [4, 7–10, 18]. Using real data from this hospital Trust, it was confirmed that the service currently used the PRN regime. The model calculated that there was an average rate of 4 injections in year 1, 3 in year 2, and 2.5 in year 3 with this regime. The service would like to change their treatment methodology to T&E in order to improve patient outcomes. However, this would mean an increase in the number of injections to 8, 6 and 4–5 in years 1, 2 and 3, respectively. Analysis of the service data indicated that there was insufficient capacity to increase the number of injections at neutral cost without other service changes. The adoption of virtual clinics seemed to have the impact of reducing the number of FU appointments at the Trust.

### Conceptualising retinal service patient pathway

The objective was to explore the clinic pathway in retinal services in order to establish what, in the experts’ opinion, were important areas for development. The pathway mapping consisted of structured interviews with retinal service nurses across a number of clinics between March and August 2015. The interviewer discussed each stage of the pathway with the interviewee taking account of the interviewee’s opinion and adjusting the pathway in ‘real time’ as comments were made.

**Fig. 1:**
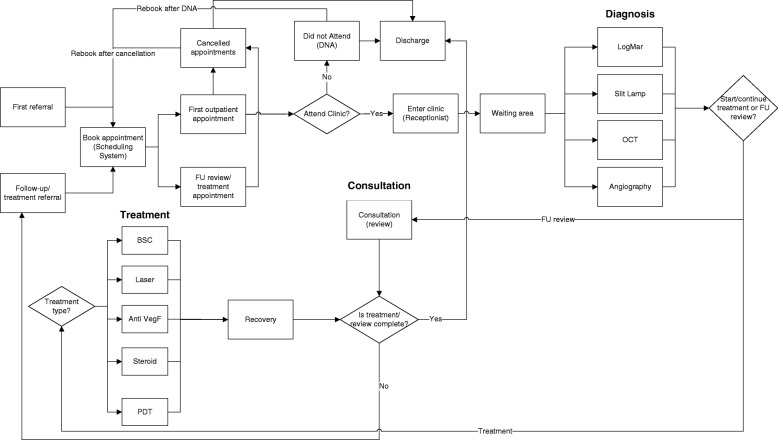
Conceptualised pathway for retinal service patients

According to the interviews, the typical care system in place in the NHS and elsewhere, for the diagnosis, initial treatment and subsequent FU/treatments in patients with eye problems comprised of a complex set of services offered in and out of hospital. Fig. 1 shows the inner workings of the retinal service pathway diagrammatically. Patients were referred (first referral) to retinal services via their General Practitioner (GP) or community optometrist and, on a small number of occasions, via accident and emergency (A&E) or outpatient services. FU evaluation and treatment would subsequently follow. Existing patients will either continue treatment (primarily with intravitreal injections) and/or continue with monitoring/assessment (follow-up review) as described in SUSTAIN study [4]. To facilitate real life setting, the Trust’s appointment booking system was incorporated into the model to ensure bookings were captured accordingly. Where a patient either cancelled or ‘did not attend’ (DNA) re-bookings were made according to the service rules.

### Patient level data analysis and forecasting

Thirty-six months of individual patient level data was obtained from Cambridge University Hospitals NHS Trust (April 2013 – March 2016). Using the statistical software R (library package *forecast*), a series of models was developed to forecast activity by patient type (nAMD, DR and RVO) broken down into “new patients” and “FUs”. The forecasted activity was then adjusted for population growth rates and collated to estimate future activity for 2016–17 (12 months forecast). When the projected activity for 2015–16 was compared with the actual activity, forecast accuracy was in the region of 90–99%.

In addition to demand forecasting, many parameters were estimated and their distributions were identified by using the *fitdistr* package in R, such as the distribution of the number of injections in years 1, 2 and 3 of treatment, waiting time for the first outpatient appointment, distribution of the number of FU assessments in each of the 3 years. Individual patients were tracked over the 36 month period and the number of times the patient was injected and monitored was counted within each of the 3 years.

### Model input parameters

Model inputs included staffing levels and salaries, staff availability, staff responsibilities, treatment pathways, arrivals (forecasted activity by new vs. follow-up attendances), percentage of patients falling into each category (nAMD, DR and RVO), costing of each service/drug/staff, treatment option visit parameters, appointment booking system parameters, clinician rota system, room/injection bed availability and weekly clinic sessions. The majority of input parameters were estimated through exhaustive analysis of patient level data, and where no data was available literature and/or expert opinion was used. See Additional file 1: Table S5 for the list of input parameters.

### Service re-design

Service delivery configurations involving increased use of community services (e.g. virtual clinics) could yield efficiency gains both operationally and financially. Operationally, it reduces unnecessary hospital attendances to ophthalmology clinics, and the need for consultations with ophthalmology doctors and nurses. Financially, treating patients in the community (i.e. within a virtual clinic setting) has a significant cost advantage over treatment in hospitals. The average cost of an outpatient follow-up assessment (or routine check-up) is around £89, whereas the same visit within a virtual clinic would cost in the region of £30–£45 [19]. This is why increased use of virtual clinics are welcomed by the Department of Health and Social Care in England, and in-line with their current policies. Virtual clinics are also welcomed by key decision makers within ophthalmology clinics, including service managers, consultants and senior nurses. It relieves the pressure for patient’s waiting for an outpatient appointment (first referral) and existing patients to receive frequency of treatment as recommended by the National Institute for Health and Care Excellence (NICE) [20]. The vast majority of research with regard to ophthalmology clinic operations and performance evaluation, has focused on quality of life of patients and the effects of AMD, DR and RVO related symptoms and social factors on the physical and psychological wellbeing of patients.

The lack of research to evaluate operational and cost performance of possible service re-designs within retinal services warrants further investigation. The discrete event simulation model we have developed here enables us to experiment any combination of service re-design configurations, i.e., the model is generic enough to run any set of scenarios.

Amongst many service configurations virtual clinic is known to be the most popular form of service re-design, which was also requested by ophthalmology (retinal service) managers, doctors and senior nurses. In addition to virtual clinics we also experiment the system under a new treatment regime. Therefore, in order to change from the existing service utilisation of the PRN to a T&E regime to improve patient outcomes, an increase in the number of injections to 8, 6 and 5 in years 1, 2 and 3, respectively would be required. Analysis of the service data showed clearly that at the time there was insufficient capacity to increase the number of injections. Projections were made as to how many appointment slots were required in order to bridge the capacity shortfall. The impact such a service change on activity, resource utilisation, costing and revenue required determination.

## Results

According to the patient level data the Trust had a total of 6520 attending patients, including 17.2%, 42.3% and 40.5% are nAMD, DR and other medical retina (MR) conditions (including 50% RVO patients, and the remaining 50% which were non-AMD/DR/RVO related), respectively.

Over the 3 year period of April 2013 – March 2016, a total of 22,129 appointments were attended, of which 42% were nAMD related (9354), 32% DR (6977) and 26% other MRE diseases including RVO (5798) (see Table 1).

**Table 1:** Breakdown of activity by attended appointments during April 2013 – March 2016

Activity	AMD	DR	MR
No. of intravitreal injections	3295	425	743
No. of intravitreal injection related FU assessments	4851	599	955
No. of laser treatments	N/A	315	63
No. of laser related FU assessments	N/A	848	191
No. of observations	1208	4790	3846
Total	9354	6977	5798

Patients under observation refer to a group of patients who had no form of treatment, hence under observation only

The 3295 injections for nAMD patients incurred 4851 FU assessments and a further 1208 assessments for patients who had no form of treatment (observation only). Approximately 69% and 66% of activity for DR and MR respectively were observation/monitoring only, and did not require any intravitreal injections.

Table 2 shows the average number of intravitreal injections and FU assessments over the 3 year data period. Patient level data in this study showed that patients received between 1 and 14 times in a year with significantly lower averages. However, the model inputs utilised distributions rather than these averages, for example, the distribution for FU assessments for observations is log-normal with mean of 1.4 and standard deviation (SD) of 0.37. According to the data, only 1% and 19% of treated and non-treated patients, respectively are discharged. Amongst those non-treated discharged patients, the vast majority are discharged at the end of year 1 and the discharge probability reduced dramatically from year 2 onwards (see Table 3 for details). In the UK, discharge from the hospital is the point at which the patient leaves the hospital and either returns home or is transferred to another facility.

**Table 2:** The average number of intravitreal injections and FU assessments over the 3 year data period

	AMD			DR			MR		
	Year of treatment			Year of treatment			Year of treatment		
	Year 1	Year 2	Year 3	Year 1	Year 2	Year 3	Year 1	Year 2	Year 3
Average no. of intravitreal injections	4	3	2.5	3.5	2.6	2.6	4	2.8	2.4
Average no. of FU assessments for injected patients	4.8	4	3.6	3.3	3.1	3.6	3.7	3.1	2.5
Average no. of FU assessments for non-treated patients (i.e. observations only)	1.7	1.6	1.4	1.4	1.4	1.2	1.4	1.4	1.1

**Table 3:** The number of discharged patients whether in treatment or under observation over 36 month’s data period broken down by patient type

	In treatment	Under observation	Total
Number of discharged patients at the end of Year 1 of treatment			
AMD	12	147	159
DR	1	330	331
MR	13	611	624
Number of discharged patients at the end of Year 2 of treatment			
AMD	16	14	30
DR	5	32	37
MR	13	58	71
Number of discharged patients at the end of Year 3 of treatment			
AMD	10	10	20
DR	1	24	25
MR	4	23	27
Total discharges	75 (1%)	1249 (19%)	1324 (20%)
Total non-discharges	950 (15%)	4256 (65%)	5206 (80%)
Total	1025 (16%)	5505 (84%)	6530 (100%)

The number of patients who had no form of treatment (ie observations only) over the data period was 5505 (Table 3). Out of this cohort the number of patients who attended in the last 2 years and were ‘observation only’ (ie persons who had no form of treatment) in the last 6 months was 894. This group of patients were referred to a nurse-led virtual clinic, where images are obtained from patients in absence of the clinician. The images were then reviewed subsequently by a clinician or other trained personnel who made disease management decisions. Extra capacity was achieved by referring 894 patients to a virtual clinic [18] in order to enable T&E regimen to be implemented.

Table 4 displays the monthly average efficiencies that could be achieved through service re-design as forecasted by the model. Column 1 figures display the ‘as is’, or current state for the service if it were to continue unchanged for each resource. The second column shows the impact of introducing a virtual clinic (with no change in current service), where 894 patients were seen. As these patients are shifted from current setting to the virtual clinic, we notice a reduction in all key metrics, e.g., number of follow-ups (from 726 to 642), and clinic session utilisation (71% to 64%). The model has included all costing and resourcing of running the virtual clinic. We also experimented where T&E is considered without the virtual clinic (third column). As expected, there is a sharp increase in the number of follow-ups, number of Anti-VEGF injections, and utilisation of resources. For instance, clinic and nurse utilisations has increased to a critical level of 85%, and without additional staff the system may not be able to cope with a slight increase in new attendances. This may not be a viable option, clearly showcasing the need to test the system with a combination of virtual clinics and T&E regime.

**Table 4:** The impact of current service against the combination of virtual clinics and treat and extend

Monthly averages	Current Service	Virtual Clinic + no change in current service	Treat and Extend + no virtual clinic	Virtual Clinic + Treat and Extend	% change
New attendances	119	119	119	119	0%
Follow-ups	726	642	812	708	−2%
Clinic session utilisation	71%	64%	85%	71%	0%
Nurse utilisation	71%	65%	82%	70%	−1%
Consultation room utilisation	53%	48%	55%	51%	−4%
Injection bed utilisation	29%	30%	40%	37%	28%
Number of Anti-VEGF injections	169	172	218	215	27%
Costing	£151,760	£153,963	£192,902	£190,200	25%
Revenue	£207,494	£207,494	£260,809	£251,664	21%
Surplus	£55,735	£53,532	£67,906	£61,464	10%

The fourth column shows the efficiencies generated comparing the current service against the combination of virtual clinics and T&E. A T&E regime in combination with a virtual clinic did not result in an increase in staff utilisation, but provided a noticeable increase in capacity with a 10% surplus. This provided the opportunity for investment in the service, with the increase in the quality of treatment and improvement in patient outcomes.

## Discussion

The ever increasing demand in medical retinal services following the introduction of new therapies has resulted in serious capacity constraints, such that some NHS clinics are failing to maintain the recommended follow-up/treatment intervals. This may have an increased risk of permanent vision loss, with huge impact on independence and quality of life of patients. It is, therefore, clear that robust and long term retinal service models that do not compromise visual outcomes, such as T&E in combination with virtual clinics, are required across NHS clinics in order to meet the needs of local populations currently and in the future. Such long-term management strategy of retinal disease in virtual clinics has been employed by a number Trusts in the UK and proven to be safe and effective if properly implemented [18].

The developed model is a new tool for forecasting and planning to support decision making in ophthalmology services. Using patient level data relating to the service, in conjunction with forecasting and simulation, enables a Trust to confidently predict the impact of making changes within the service. The flexibility of the model enables the impact of a wide range of scenarios to be tested (around several thousand scenarios can be tested). A potential solution (T&E in combination with virtual clinic) is presented here that is shown to improve quality, effectiveness and productivity and thus increase capacity. Despite the increase in the number of injections (with T&E), the impact on resource utilisation is negligible (almost 0%) and in some cases below 0%.

Spreadsheet based models commonly use averages, for example the average number of patients expected each day followed by the average number of patients who will be diagnosed with nAMD followed by the average number of injections in the first year. The problem with using averages in this way may lead to wildly incorrect predictions, commonly known as the ‘error of averages’ (Salvage, 2009) [21]. Arguably when considering services that have a turnover of many millions of pounds it is very important to be accurate.

Unlike spreadsheet models, the power of discrete event simulation in general is the ability to capture variability. For example, some guidelines assume that anti-VEGF treatments are injected on average of 7 times in the first year, although patient level data analysis in this study showed that there were 1–14 intravitreal injections in a year with significantly lower averages. As such assuming that all patients received an average of 7 injections per year could be misleading.

The choice of using simulation methods as opposed to analytical methods was partly dictated by the complexity of the pathway and the ease of use for end-users. In this respect, the need to track individual patient journeys (or trajectories) through the care system, the ability to capture the complex web of interactions of patients going through the diagnosis stage to various forms of treatment (including FUs) and the need to model notions of limited availability of resources (such as staff, beds, rooms and equipment) have motivated us to select discrete event simulation.

Instead of using averages our model uses each hospital Trusts’ anonymised patient level data to track individual patients over a 36-month period. It counts the number of times a patient is treated and assessed in years 1, 2 and 3. It then uses statistical algorithms to establish the distribution, for example, treatment frequency for each year, for example log normal distribution with a mean and SD. The model is then run hundreds of times for thousands of patients where the distribution determines the number of times a patient is injected, as opposed to using an average of 7 injections per year for each patient. It is because the model pays close attention to these details, that it is able to create validated reports that are up to 95% accurate.

A constraint on modelling analysis is the recognition of essential resources that are required in order for an event to happen. For example, it is not possible for a clinic to start without a clinician. The model only starts a clinic when all the resources required are available. Intravitreal services are complex with many hundreds of variables/constraints. The ability to deal with these complex constraints simply does not exist in excel or any other modelling approaches. Using the model it is possible to show what happens if there is a variation in the availability of resources/constraints such as the increase or reduction in the number of clinic rooms available, or the effect of an absent clinician. Given the level of complexity and value of intravitreal services, it is extremely important to ensure that model outputs are robust and accurate.

The National Institute for Health and Care Excellence (NICE) has recognised the methodology used in the model as a valid way of simulating complex patient pathways [22]. To the best of our knowledge, this model is currently the only simulation based planning tool for retinal service managers. Other such programmes or further development would be welcome.

Re-design of clinical services based on such programmes, nevertheless, requires investment, although such investment is outweighed by the potential increases in surplus generated from efficient service re-design. More importantly, optimum service re-design also leads to better quality of care that meet patients’ needs, as well increased productivity by maximising the use of experienced clinicians. Furthermore, significant cost reductions are expected in the long-term as more patients retain their vision with better clinical outcomes due to the T&E or other similar regimen.

The authors are aware of the many difficulties that are faced in the planning, approval, and implementation of new retinal services. Changes can be introduced without proper consideration of the impact on the service, and as a result most business cases are inaccurate [12]. Frequently clinicians working within retinal services know how they would like to improve the service they deliver, but lack the expertise to frame those improvements in a manner that is acceptable to health service executives and managers who hold finance budget. The model was developed in conjunction with retinal specialists, specifically to address these issues. It is designed to allow ‘non-experts’ to test change on the pathway within the validated simulation. The simulation will present the impact of changes in a way that can be easily understood by both the healthcare executives and the pathway specialists. The intention is that this tool will facilitate service planning and decision making and speed up the pace of change in the retinal pathway.

The tool allows decision makers to develop a better understanding of key performance metrics associated with activity, cost implications and resource utilisation. The ease of use of the tool with relevant sets of exported results means that senior decision makers can be more proactive and confident with evidence based approach in re-designing their care pathway in finding the most efficient and effective delivery of care to patients with problems in the eye.

## Conclusions

In conclusion, the model allows the use of pathway planning to improve clinic efficiency and patient outcomes without necessarily increasing costs in the long-term.

## Additional file

Additional file 1: BMC HSR Data for simulation model. Table S5. Data used for the simulation model. Table providing details of parameters used in the simulation model including the source, distribution type and the value entered in the model. (DOCX 19 kb)

## Abbreviations

A&E: Accident & Emergency
AMD: Age Related Macular Degeneration
anti-VEGF: anti-Vascular Endothelial Growth Factor
DNA: Did Not Attend
DR: Diabetic Retinopathy
FU: Follow-up
GP: General Practitioner
MR: Medical Retina
nAMD: neovascular Age Related Macular Degeneration
NHS: National Health Service
NICE: National Institute for Health and Care Excellence
PRN: Pro Re Nata
RVO: Retinal Vein Occlusion
SD-OCT: Spectral Domain Optical Coherence Tomography
T&E: Treat & Extend
