# Supplementary material for: A simulation tool for better management of retinal services
Source: BMC Health Serv Res. 2018 Oct 4;18:759. doi: 10.1186/s12913-018-3560-5 (PMC6172737; doi:10.1186/s12913-018-3560-5)
Supplement: Supplementary file 1 — BMC HSR Data for simulation model. Table S5. Data used for the simulation model. Table providing details of parameters used in the simulation model including the source, distribution type and the value entered in the model. (DOCX 19 kb) [file 12913_2018_3560_MOESM1_ESM.docx]

|  | **Estimate** | **Distribution** | **Reference** | |
| --- | --- | --- | --- | --- |
| **Patient Type** | | | | |
| Proportion of patients with Age Related Macular Degeneration (AMD) | User specified | Multinomial | Local data | |
| Proportion of patients with Diabetic Retinopathy (DR) | User specified | Multinomial | Local data | |
| Proportion of patients with Retinal Vein Occlusion (RVO) | User specified | Multinomial | Local data | |
| Proportion of patients under Observation | User specified | Multinomial | Local data | |
| **Demand** | | | | |
| Monthly demand for first appointments | Forecasted | N/A | Local data | |
| Monthly demand for follow-up appointments | Forecasted | N/A | Local data | |
| Monthly ‘did not attends’ | Forecasted | N/A | Local data | |
| **Diagnostic kits** | | | | |
| **Percentage use of the following diagnostic kits:** |  |  |  | |
| LogMAR chart | 100% | Multinomial | Local data | |
| Slit Lamp | 100% | Multinomial | Local data | |
| Angiography | 20% | Multinomial | Local data | |
| OCT | 70% | Multinomial | Local data | |
| **Treatment** | | | | |
| **AMD related treatment regime** |  |  |  | |
| Lucentis (Ranibizumab) | 46% | Multinomial | Local data | |
| Observation | 54% | Log normal | Local data | |
| **RVO related treatment regime** |  |  |  | |
| Laser | 2% | Multinomial | Local data | |
| Lucentis (Ranibizumab) | 7% | Multinomial | Local data | |
| Observation | 91% | Log normal | Local data | |
| **DR related treatment regime** |  |  |  | |
| Laser | 4% | Multinomial | Local data | |
| Lucentis (Ranibizumab) | 12% | Multinomial | Local data | |
| Observation | 84% | Log normal | Local data | |
| **The average number of follow-up visits for those in intravitreal injection treatment** |  | | | |
|  | **AMD** |  |  | |
| First year of treatment | 5 | Log normal | Local data | |
| Second year of treatment | 4 | Log normal | Local data | |
| Third year of treatment | 4 | Log normal | Local data | |
|  | **DR** |  |  | |
| First year of treatment | 4 | Log normal | Local data | |
| Second year of treatment | 3 | Log normal | Local data | |
| Third year of treatment | 3 | Log normal | Local data | |
|  | **RVO** |  |  | |
| First year of treatment | 4 | Log normal | Local data | |
| Second year of treatment | 3 | Log normal | Local data | |
| Third year of treatment | 3 | Log normal | Local data | |
| **Percentage of patients in:** | | | | |
| First year of treatment | 72% | Multinomial | Local data | |
| Second year of treatment | 18% | Multinomial | Local data | |
| Third year of treatment | 10% | Multinomial | Local data | |
| **Percentage of patients discharged**  **at the end of:** | | | | |
| First year of treatment | 5% | Multinomial | Local data | |
| Second year of treatment | 2% | Multinomial | Local data | |
| Third year of treatment | 1% | Multinomial | Local data | |
| **Resources** | | | | |
| **Rooms** | | | | |
| Consultation room | 4 | Fixed | Local data | |
| Injection Bed | 1 | Fixed | Local data | |
| Theatre | 1 | Fixed | Local data | |
| **Staff** | | | | |
| Number of Consultant clinics per week | 33 | Fixed | Local data | |
| Nurses | 4 | Fixed | Local data | |
| Healthcare Assistants | 5 | Fixed | Local data | |
| Technicians | 2 | Fixed | Local data | |
| Photographer | 1 | Fixed | Local data | |
| **Diagnostic kits** | | | | |
| Slit Lamp | 2 | Fixed | Local data | |
| OCT | 2 | Fixed | Local data | |
| Angiography | 1 | Fixed | Local data | |
| **Revenue and Costing** | | | | |
| **Revenue** | | | | |
| 1st Outpatient Appointment | £112 | Fixed | National Tariff Payment (2016/2017)^21^ | |
| Follow-up assessment | £63 | Fixed | National Tariff Payment (2016/2017)^21^ | |
| Best Supportive Care | £112 | Fixed | National Tariff Payment (2016/2017)^21^ | |
| Laser | £292 | Fixed | National Tariff Payment (2016/2017)^21^ | |
| Photo Dynamic Therapy | £107 | Fixed | National Tariff Payment (2016/2017)^21^ | |
| **Costing** | | | | |
| Cost of administering injections | £140 | Fixed | | (NICE, 2013)^22^ |
| Avastin (Bevacizumab) | £120 | Fixed | | (MIMS)^23^ |
| Lucentis (Ranibizumab) | £742.17 | Fixed | | (MIMS)^23^ |
| Eylea (Aylibercept) | £816 | Fixed | | (MIMS)^23^ |
| Ozurdex (Dexamethasone) | £870 | Fixed | | (MIMS)^23^ |
| Kenolog (Triamcinolone) | £1.49 | Fixed | | (MIMS)^23^ |
| Illuvien (Fluocinolone Acetonide) | £5500 | Fixed | | (MIMS)^23^ |
| **Salary - Hourly Cost** | | | | |
| Consultant | £48.64 | Fixed | PSSRU (2013) | |
| Optometrist | £17.66 | Fixed | PSSRU (2013) | |
| Nurse | £14.27 | Fixed | PSSRU (2013) | |
| Optician | £17.66 | Fixed | PSSRU (2013) | |
| Technician | £14.27 | Fixed | PSSRU (2013) | |
| Photographer | £17.66 | Fixed | PSSRU (2013) | |
| Healthcare Assistant | £9.85 | Fixed | PSSRU (2013) | |

Table 5. Data used for the simulation model. OCT = Optical Coherence Tomography; AMD = Age related Macular Degeneration; DR = Diabetic Retinopathy; RVO = Retinal Vein Occlusion.
